# Label‐free imaging of intracellular structures in living mammalian cells via external apodization phase‐contrast microscopy

**DOI:** 10.1111/febs.70286

**Published:** 2025-10-17

**Authors:** Hiroshi Ohno, Takenori Nishimura, Kenta Kainoh, Yoshitaka Ohashi, Naoko Onodera, Mayuko Kano, Lay Nurhana Sari, Masato Masuda, Yoshiaki Tamura, Teppei Nishino, Yusuke Hayashi, Yusuke Yamamoto, Shin‐Ichiro Takahashi, Yuta Mishima, Yosuke Yoneyama, Yoshinori Takeuchi, Motohiro Sekiya, Takashi Matsuzaka, Takafumi Miyamoto, Hitoshi Shimano

**Affiliations:** ^1^ Department of Endocrinology and Metabolism Institute of Medicine, University of Tsukuba Japan; ^2^ Department of Animal Sciences and Applied Biological Chemistry, Graduate School of Agricultural and Life Sciences The University of Tokyo Japan; ^3^ Department of Information Sciences and Arts, Faculty of Information Sciences and Arts Toyo University Saitama Japan; ^4^ Laboratory of Integrative Oncology National Cancer Center Tokyo Japan; ^5^ Transborder Medical Research Center University of Tsukuba Japan; ^6^ Department of Clinical Medicine Institute of Medicine, University of Tsukuba Tsukuba Japan; ^7^ Institute of Research Tokyo Medical and Dental University Tokyo Japan; ^8^ Center for Cyber Medicine Research University of Tsukuba Tsukuba Japan

**Keywords:** biomolecular condensates, cellular organization, external apodization phase‐contrast microscopy, lipid droplets, mitochondria dynamics

## Abstract

Developing techniques to visualize intracellular structures, which influence the spatiotemporal functionality of biomolecules, is essential for elucidating mechanisms governing cellular behavior. In this study, we demonstrate that label‐free external apodized phase‐contrast (ExAPC) microscopy serves as a valuable tool for the simultaneous observation of various intracellular structures with high spatiotemporal resolution, while successfully mitigating halo artifacts. Additionally, through quantitative analysis of images obtained by combining ExAPC microscopy with fluorescence microscopy, we identified distinct heterogeneities in biomolecular condensates, lipid droplets, and mitochondria. Our findings highlight the potential of ExAPC microscopy to provide detailed insights into alterations in intracellular structures associated with diverse cellular processes, corroborating the existing knowledge and potentially contributing to the discovery of previously unknown cellular mechanisms.

## Graphical abstract

Advanced techniques to visualize the spatiotemporal functionality of biomolecules are essential for elucidating mechanisms governing cellular behavior. External apodization phase‐contrast (ExAPC) microscopy enables label‐free visualization of diverse intracellular structures in living mammalian cells. By enhancing phase‐contrast imaging through optical apodization, ExAPC reveals fine subcellular details, such as lipid droplets and organelle contacts, without fluorescent labels. This method provides a powerful, non‐invasive tool to study cellular organization and dynamics, supporting broad applications in cell biology and biomedical research.

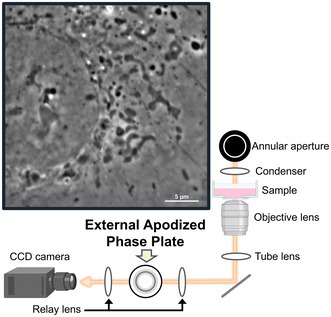

**Abbreviations**
BCLs: biomolecular condensate‐like structures
DRP1: dynamin‐related protein 1
ELL: endosome‐ and lysosome‐like structure
ER: endoplasmic reticulum
ExAPC: external apodized phase contrast
FCCP: carbonylcyanide‐p‐trifluoromethoxyphenylhydrazone
GFP: green fluorescent protein
hiPSC: human‐induced pluripotent stem cell
IGF: insulin‐like growth factor
IRS‐1: insulin receptor substrate 1
LD: lipid droplet
MFN: mitofusin
ODT: optical diffraction tomography
OPA1: optic atrophy‐1
ROS: reactive oxygen species
TMRM: tetramethylrhodamine methyl ester
ΔΨm: mitochondrial membrane potential

## Introduction

Cells harbor intracellular structures called organelles that perform many diverse functions. The characteristic dimensions, quantities, and spatial arrangements of each organelle lead to the overall morphological attributes of the cell, termed cellular organization [1]. Many researchers have suggested that cellular organization, in concert with the functional attributes of cellular organelles, plays a pivotal role in determining diverse cellular behaviors [2, 3, 4, 5].

To understand the significance of cellular organization and its influence on various cellular behaviors, it is essential to visualize and clarify the intricate features of intracellular structures. Fluorescence imaging has become a fundamental tool for this purpose [6, 7]. This technique enables detailed visualization of intracellular structures by employing fluorescent markers that specifically localize to those structures. Advances in molecular genetics have made fluorescent labeling more accessible, offering advantages such as speed, specificity, and applicability across various model organisms. However, fluorescence imaging has limitations, including photobleaching and phototoxicity [8]. In response, recent attention has shifted toward label‐free imaging techniques, which exploit the optical contrast generated by light interactions with cells [9]. This approach avoids the need for labeling and generates cellular images in a state closer to the native environment.

**Fig. 1:**
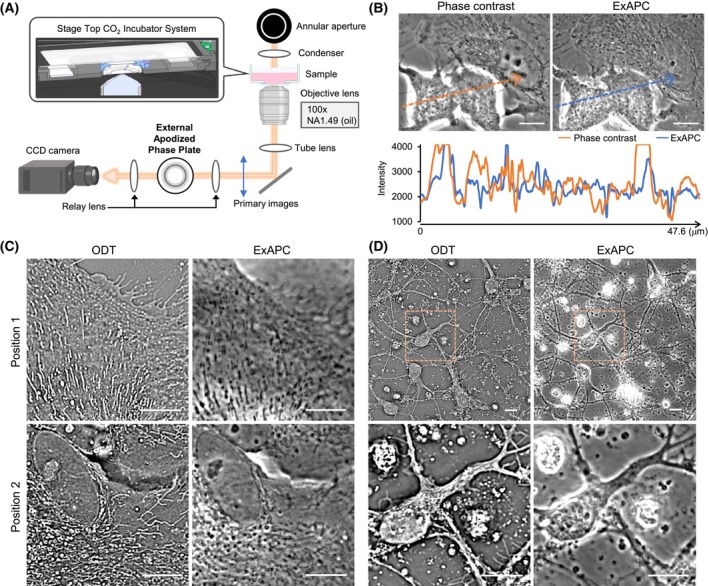
Visualization of cellular organization utilizing ExAPC microscopy. (A) Schematic representation of the external apodized phase‐contrast (ExAPC) microscope used in this study. Illustration created with BioRender.com. (B) Phase‐contrast and ExAPC microscopy images of live A549 cells. Arrows indicate traces of the line scan. (C) Optical diffraction tomography (ODT) and ExAPC microscopy images of live primary astrocytes. (D) ODT and ExAPC microscopy images of live primary neurons. All scale bar: 10 μm.

Several innovative label‐free imaging techniques have been developed [10, 11, 12], including apodized phase‐contrast microscopy [13, 14]. In apodized phase‐contrast microscopy, the sample is positioned in the front focal plane of the objective lens and illuminated through an annular aperture. The incident light passes through an apodized phase ring surrounded by two light‐attenuating rings, inducing a phase shift. The diffracted light passing through these attenuating rings is minimized, reducing halo artifacts [15]. Although commercially available objective lenses with an apodized phase plate exist, high‐magnification lenses suitable for observing intracellular structures are not accessible. Nevertheless, the value of advanced visualization techniques lies in ensuring their accessibility to the broader scientific community. To address this gap, we employed an external apodized phase‐contrast (ExAPC) microscopy method, in which an apodized phase plate is placed outside the objective lens (Fig. 1A). This approach overcomes the inherent limitations of commercially available lenses and facilitates the simultaneous imaging of multiple organelles while significantly reducing halo artifacts compared to conventional phase‐contrast microscopy (Fig. 1B).

In this study, we demonstrated the utility of the ExAPC microscopy technique for observing cellular organization. Furthermore, through a detailed examination of biomolecular condensates, lipid droplets, and mitochondria that constitute cellular organization, we revealed the diversity in their behaviors and characteristics.

## Results

### Comparison of ExAPC microscopy and ODT for imaging cellular organization

ExAPC microscopy is a two‐dimensional phase‐imaging technique, whereas optical diffraction tomography (ODT) as a technique to visualize intracellular structures enables three‐dimensional phase imaging and has recently garnered attention. A comparison between ExAPC microscopy and ODT showed that both techniques provide comparable results for visualizing the morphology of intracellular structures (Fig. 1C). A major difference between these techniques lies in their temporal resolution: ExAPC microscopy achieves subsecond imaging intervals (Movie S1), whereas obtaining comparable spatial and temporal resolutions with ODT, which requires Z‐axis imaging, remains challenging [16, 17]. Despite this, ODT offers the advantage of imaging thicker specimens, a task that presents limitations for ExAPC microscopy (Fig. 1D). These results indicate that the choice between ExAPC microscopy and ODT should depend on experimental objectives, as both modalities offer complementary advantages in advancing our understanding of cellular organization.

### Visualization of cellular organization in live cells using ExAPC microscopy

**Fig. 2:**
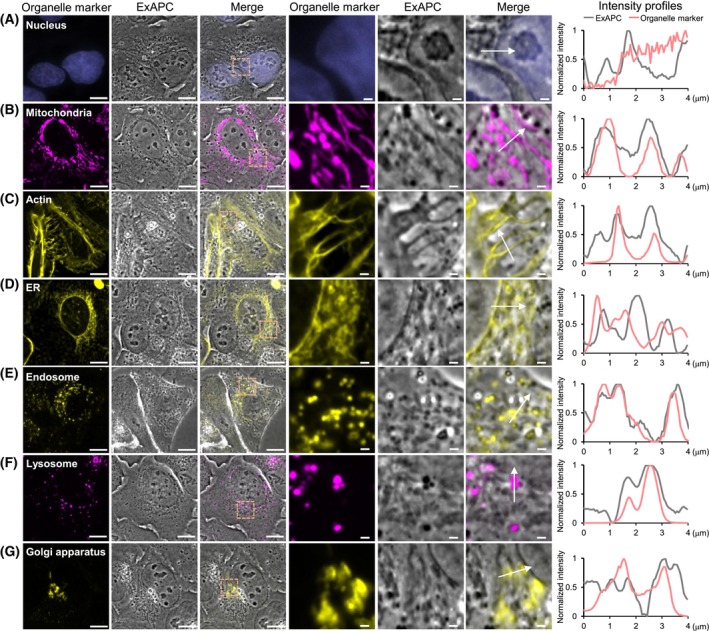
Visualization of cellular organization utilizing ExAPC microscopy. Comparison of organelle visualization and intensity profiles using ExAPC and epifluorescence microscopy with the indicated organelle markers. (A) Nucleus: Hoechst 33342. (B) Mitochondria: MitoTracker Red CMXRos. (C) Actin: Lifeact‐EYFP. (D) Endoplasmic reticulum (ER): EYFP‐Cb5. (E) Endosome: ECGreen. (F) Lysosomes: LysoTracker Red DND‐99. (G) Golgi apparatus: EYFP‐sGOLGB1. The intensities obtained by ExAPC microscopy are shown as absolute values after subtracting 1 from the normalized value. Arrows indicate traces of the line scan. Scale bar: 10 μm, scale bar in inset: 1 μm.

**Fig. 3:**
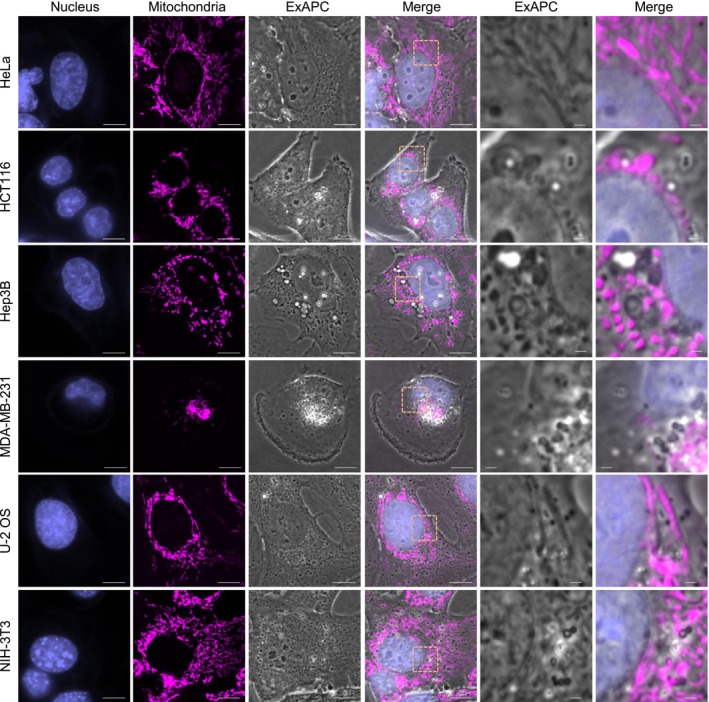
Various cell lines visualized by ExAPC microscopy. Representative images of the indicated cell lines acquired by ExAPC microscopy are shown. Nucleus: Hoechst 33342; mitochondria: MitoTracker Red CMXRos. Scale bar: 10 μm, Scale bar in inset: 1 μm.

The ExAPC microscopy system facilitates the label‐free visualization of diverse intracellular structures within living cells. Nevertheless, the unambiguous identification of these structures based solely on the images obtained remains a substantial challenge. To overcome this limitation, we combined ExAPC microscopy with fluorescence imaging to accurately identify organelles detectable using the ExAPC system. Structures such as nuclei, nucleoli, and mitochondria, characterized by refractive indices distinct from the cytoplasm [17], were effectively visualized through ExAPC microscopy (Fig. 2A,B). Additionally, actin filaments were discernible using this approach (Fig. 2C). While the endoplasmic reticulum (ER) was difficult to detect in proximity to the nucleus, peripheral regions of the cell revealed structures partially colocalizing with ER‐specific markers (Fig. 2D). Furthermore, numerous vesicular structures, positively stained with markers for endosomes and lysosomes, were identified using the ExAPC microscopy system (Fig. 2E,F). The average size of these vesicles was calculated to be 0.177 ± 0.168 μm^2^ (*n* = 1239), consistent with previously reported dimensions for these organelles [18, 19]. Therefore, these vesicular structures were designated as ‘endosome‐ and lysosome‐like structure (ELL)’. Conversely, the Golgi apparatus, which exhibits minimal refractive index contrast with the cytoplasm, remained challenging to detect using ExAPC microscopy alone (Fig. 2G). For such organelles, fluorescence imaging is indispensable when used in conjunction with ExAPC microscopy. Moreover, we demonstrated the capability of ExAPC microscopy to visualize intracellular structures across various live‐cell lines, including human cancer cell lines (HeLa, HCT116, Hep3B, MDA‐MB‐231, U‐2 OS) and mouse fibroblasts (NIH‐3 T3), highlighting its broad applicability and potential utility in diverse biological contexts (Fig. 3).

**Fig. 4:**
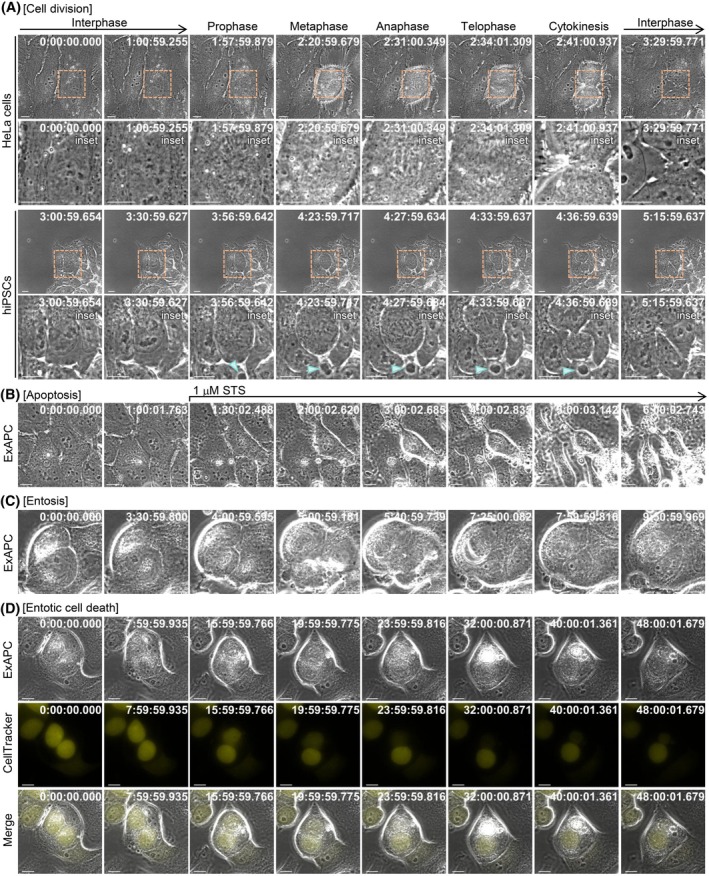
Visualization of diverse cellular behaviors using ExAPC microscopy. Representative images of the indicated cellular behaviors acquired by ExAPC microscopy are shown. (A) Cell division of HeLa cells and human induced pluripotent stem cells (hiPSCs). Acquisition interval: 1 min·frame^−1^. Arrowhead: Biomolecular condensate‐like structure; see Fig. 5. (B) HeLa cells were treated with 1 μm staurosporine (STS) at frame 14. Acquisition interval: 5 min·frame^−1^. (C) Entosis of ZR75‐1 cells. Acquisition interval: 1 min·frame^−1^. (D) ZR75‐1 cells stained with CellTracker Green CMFDA and unstained ZR75‐1 cells were cocultured. Acquisition interval: 1 min·frame^−1^. Scale bar: 10 μm (main and inset). Time: h:mm:ss.ms.

Reliable information on the dynamics of cellular organization offers insights into the mechanisms driving various cellular behaviors. ExAPC microscopy utilizes an apodization method to effectively reduce halo artifacts (Fig. 1B). As a result, ExAPC microscopy allowed us to capture intricate details of changes in cellular organization during the cell division of HeLa cells and human induced pluripotent stem cells (hiPSCs) (Fig. 4A and Movies S2, S3). While complete removal of halos was not always achievable—particularly during cell rounding and thickening associated with apoptosis (Fig. 4B and Movie S4) or entosis (Fig. 4C and Movie S5)—we were still able to observe intracellular structures clearly in the unaffected regions. Additionally, the ExAPC microscope enabled stable long‐term imaging of these cellular behaviors (Fig. 4D). These findings collectively highlight both the practical advantages and technical considerations of ExAPC microscopy.

### ExAPC microscopy‐based analysis of biomolecular condensates and their analogous structures

Fluorescence microscopy delineates cellular organization by utilizing intracellular structure‐specific fluorescent markers. In contrast, ExAPC microscopy converts the phase shift induced by light traversing the cell into an amplitude shift, resulting in a variation in image contrast. Unlike fluorescence microscopy, ExAPC microscopy simultaneously visualizes all intracellular structures that cause phase shifts within cells without the need for staining.

**Fig. 5:**
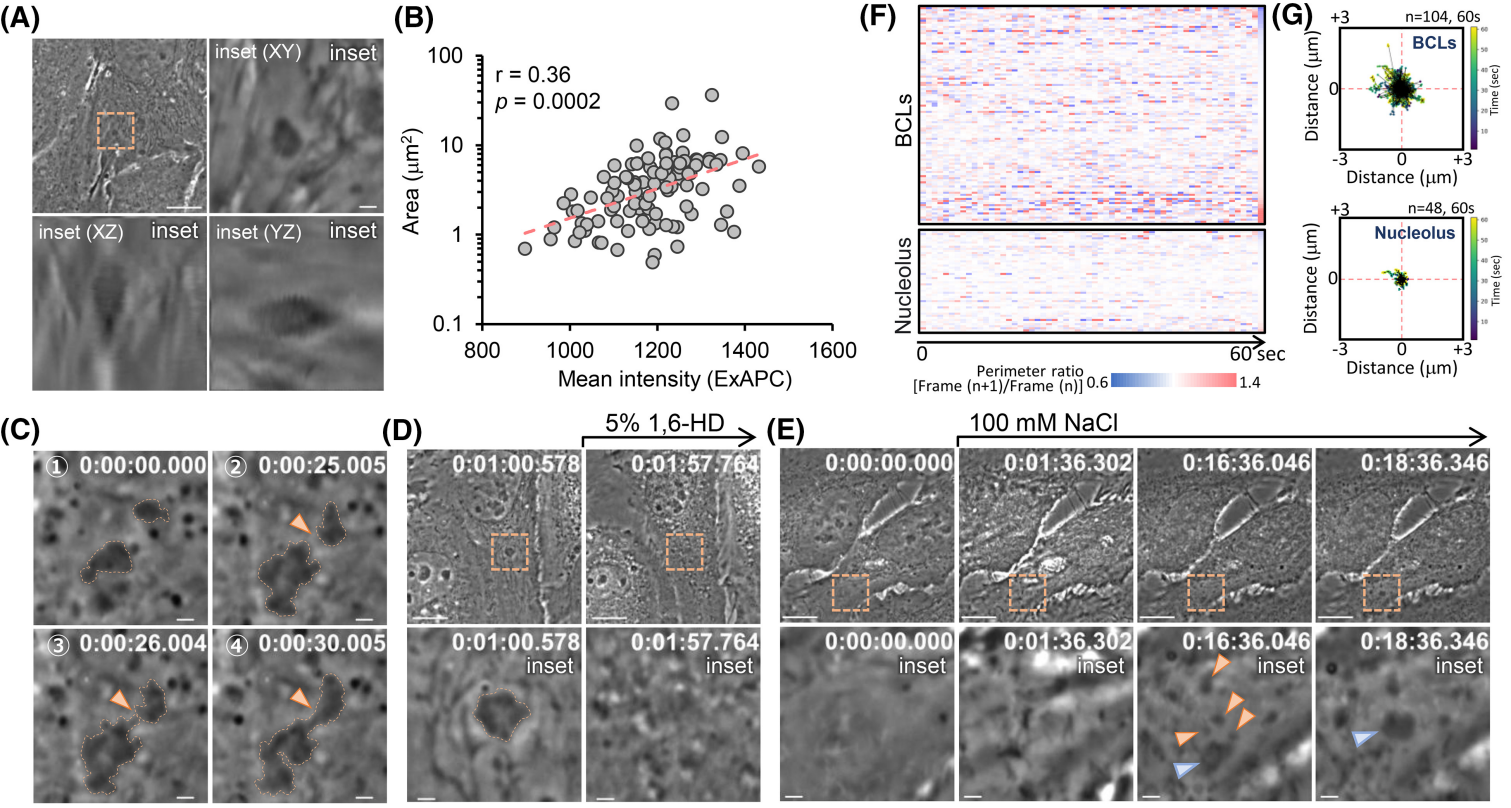
Visualization of BCLs via ExAPC microscopy. (A) Z‐stack imaging of biomolecular condensate‐like structure (BCL) in HeLa cells. Z‐Step: 0.097 μm. Scale bar: 10 μm; scale bar in inset: 1 μm. (B) Relationship between the area and average intensity of BCLs observed in HeLa cells. *n* = 104 from 45 cells. (C) Time‐lapse imaging showing the fusion of the two BCLs in HeLa cells. Arrowhead indicates the site where the BCLs undergo fusion. Acquisition interval: 1 s·frame^−1^. Scale bar: 1 μm. (D) BCL‐positive HeLa cells were treated with 5% 1,6‐hexanediol (1,6‐HD) at Frame 61. Acquisition interval: 1 s·frame^−1^. Scale bar: 10 μm; scale bar in inset: 1 μm. (E) HeLa cells were treated with 100 mm sodium chloride (NaCl) at Frame 61. Acquisition interval: 1 s·frame^−1^. Scale bar: 10 μm; scale bar in the inset: 1 μm. The BCLs indicated by orange arrowheads were fused with the BCL indicated by the blue arrowhead. (F) Heatmaps of the perimeter ratios of BCLs and nucleoli in HeLa cells. Each row represents a single BCL (*n* = 104) or the nucleolus (*n* = 48). (G) Random motions of BCLs and the nucleolus in HeLa cells. The plots show BCLs and nucleoli over 60 s, starting at the center. The analyzed BCLs and nucleolus are the same as that in F. Time: h:mm:ss.ms.

Using ExAPC microscopy, we examined live HeLa cells and identified highly dynamic and well‐defined spherical structures within the cytoplasm (Fig. 5A and Movie S6). These structures were present in 16.3% (45 of 276 cells) of HeLa cells. A similar structure was observed in hiPSCs (Fig. 4A and Movie S3). There was a weak correlation between the area and mean intensity of the structures observed in HeLa cells, suggesting that these structures are composed of biomolecules with varying refractive indices (Fig. 5B). Time‐lapse imaging revealed that these structures underwent fusion events (Fig. 5C) and treatment with 1,6‐hexanediol (1,6‐HD), an agent known to dissolve liquid phase‐separated biomolecular condensates [20], resulted in their disappearance (Fig. 5D). Furthermore, subjecting HeLa cells to hyperosmotic stress, which is known to induce the formation of biomolecular condensates [21], resulted in the appearance of similar structures in the cytosol (Fig. 5E). Based on these findings, we defined these structures as biomolecular condensate‐like structures (BCLs). The BCLs displayed dynamic changes in their perimeter (Fig. 5F) and localization (Fig. 5G) compared to the nucleolus, the major biomolecular condensate in the nucleus [22]. Thus, the mechanisms regulating the structural stability and localization of BCLs may differ from those governing the nucleolus.

**Fig. 6:**
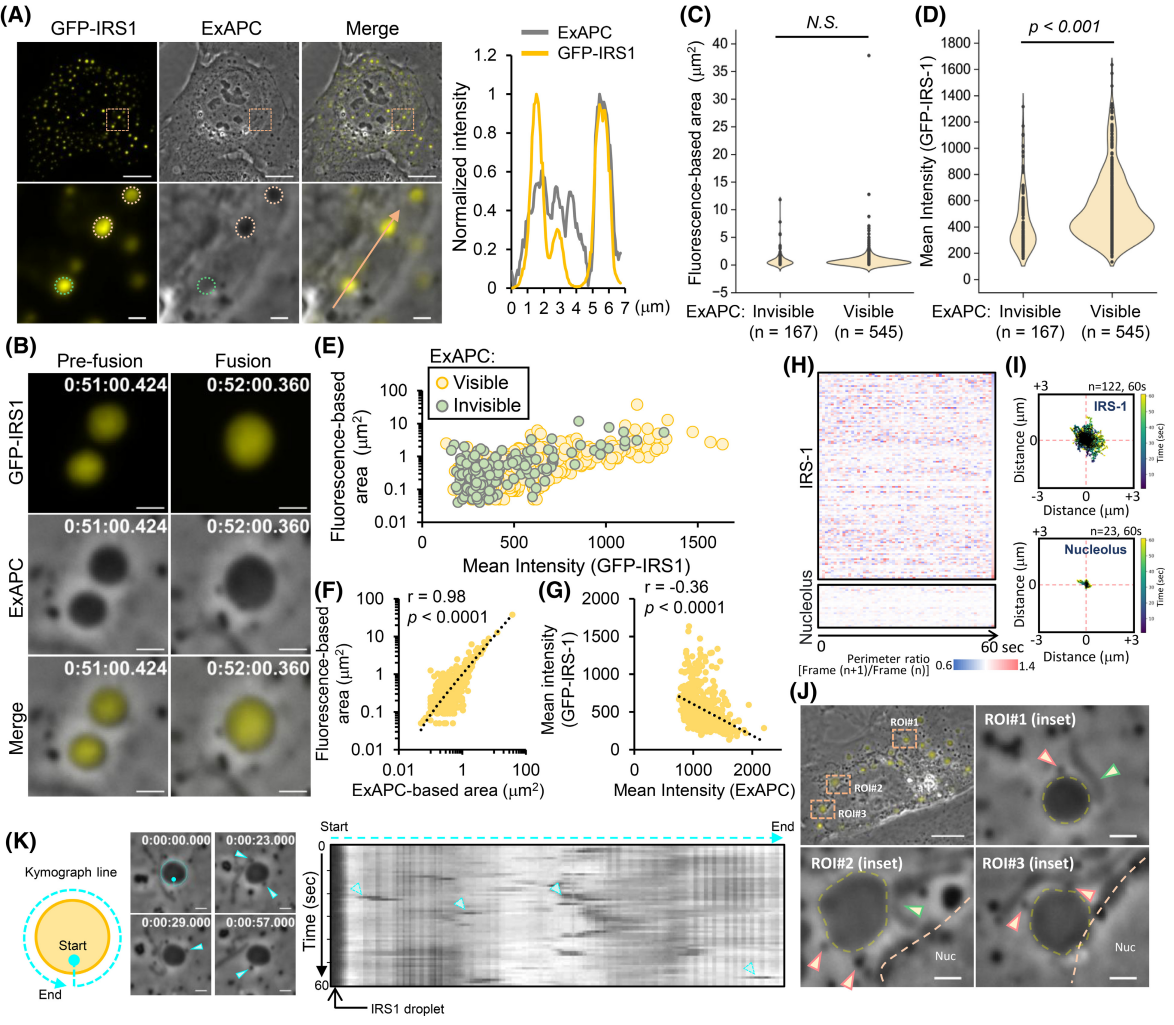
Visualization of IRS‐1 droplet via ExAPC microscopy. (A) Representative images of U‐2 OS cells expressing GFP‐tagged insulin receptor substrate 1 (GFP‐IRS1). Line scan profiles of intensity in the merged inset is shown. The intensities obtained by ExAPC microscopy are shown as absolute values after subtracting 1 from the normalized value. The IRS‐1 droplet outlined with an orange dashed line is visible in the ExAPC image, whereas the one outlined with a green dashed line is invisible. Arrow indicates traces of the line scan. Scale bar: 10 μm; scale bar in inset: 1 μm. (B) Time‐lapse imaging showing the fusion of two IRS‐1 droplets in U‐2 OS cells expressing GFP‐IRS‐1. Acquisition interval: 1 min·frame^−1^. Scale bar: 1 μm. (C and D) Comparison of the area (C) and mean fluorescence intensity (D) of IRS‐1 droplets in GFP‐IRS‐1‐expressing U‐2 OS cells that were either visible or invisible under ExAPC microscopy. *p*: Unpaired *t*‐tests. NS, statistically nonsignificant. (E) Relationship between the IRS‐1 droplet area and the mean intensity of IRS‐1 in U‐2 OS cells expressing GFP‐IRS‐1, as measured in C, D. (F and G) Relationships between the area of IRS‐1 droplets measured from ExAPC and epifluorescence images (F) and the mean intensity of IRS‐1 droplets measured from ExAPC and epifluorescence images (G). *n* = 545 from 15 U‐2 OS cells expressing GFP‐IRS‐1. (H) Heatmaps of the perimeter ratio of IRS‐1 droplets to nucleoli in 9 GFP‐IRS‐1‐expressing U‐2 OS cells. Each row represents a single IRS‐1 droplet (*n* = 122) or a nucleolus (*n* = 23). (I) Random motion of IRS‐1 droplets and nucleoli in U‐2 OS cells expressing GFP‐IRS‐1. The plots show IRS‐1 droplets and nucleoli over 60 s, starting at the center. The analyzed BCLs and nucleolus are the same as that in H. (J) Interaction of IRS‐1 droplets with various organelles in GFP‐IRS‐1‐expressing U‐2 OS cells. Green arrowheads indicate mitochondria in contact with IRS‐1 droplets, and orange arrowheads indicate endosome‐ and lysosome‐like structure (ELLs) in contact with IRS‐1 droplets. A representative image from three independent experiments is shown, all of which consistently showed such contacts. Scale bar: 10 μm; scale bar in the inset: 1 μm. (K) Kymograph showing the interaction between the IRS‐1 droplets and ELLs (arrowhead). A representative image from three independent experiments is shown. Scale bar: 1 μm. Time: h:mm:ss.ms.

To further validate the capability of ExAPC microscopy in observing biomolecular condensates, we focused on insulin receptor substrate 1 (IRS‐1). IRS‐1 is a key protein in the regulation of insulin/insulin‐like growth factor (IGF) signaling [23, 24, 25], and the phase separation of IRS‐1 promotes the formation of the insulin/IGF signalosome, also known as the IRS‐1 droplet [26]. Following the exogenous expression of green fluorescent protein (GFP)‐fused IRS‐1 in U‐2 OS cells, we observed the formation of IRS‐1 droplets [26] using both epifluorescence and ExAPC imaging (Fig. 6A). Additionally, ExAPC microscopy revealed the fusion of IRS‐1 droplets at various intracellular locations (Fig. 6B; Movie S7). Notably, 23.5% (167 of 712) of IRS‐1 droplets were detectable via epifluorescence imaging, whereas no corresponding structures were visible in the ExAPC images (Fig. 6A). Although the IRS‐1 droplets did not differ significantly in size between those visible and those invisible to ExAPC microscopy, the fluorescence intensity of droplets not visible in ExAPC imaging was significantly lower (Fig. 6C,D). This suggests that the quantity of IRS‐1 within IRS‐1 droplets may influence their refractive index. Nevertheless, determining the visibility of IRS‐1 droplets via ExAPC microscopy based solely on fluorescence intensity proved challenging (Fig. 6E).

We then focused on IRS‐1 droplets observable by both fluorescence and ExAPC microscopy. The median size of IRS‐1 droplets was determined to be 0.39 μm^2^ [Interquartile Range (IQR): 0.2–0.77 μm^2^] by epifluorescence imaging and 0.47 μm^2^ (IQR: 0.26–0.83 μm^2^) by ExAPC imaging, showing a strong correlation between the two methods (Fig. 6F). However, the intensity of IRS‐1 droplets in epifluorescence imaging weakly correlated with that observed by ExAPC microscopy (Fig. 6G). These results suggest that variations in the quantity and quality of molecules constituting the IRS‐1 droplet may influence the refractive index. Time‐lapse imaging revealed dynamic changes in both the perimeter and localization of IRS‐1 droplets, similar to those seen in BCLs (Fig. 6H,I). Furthermore, we used the multiplexing capabilities of ExAPC microscopy to investigate the interactions between IRS‐1 droplets and organelles. Consistent with previous findings [27], we observed interactions between IRS‐1 droplets and nuclei, mitochondria, and ELLs (Fig. 6J). Notably, interactions with ELLs occurred at high frequencies, with some lasting less than 1 s (Fig. 6K). The interactions between IRS‐1 droplets and organelles, occurring at various locations and over differing durations, may be critical for optimizing IRS‐1‐mediated signal transduction.

### Dissection of lipid droplet formation using ExAPC microscopy

Lipid droplets (LDs) are increasingly recognized as intracellular organelles with diverse functions beyond energy storage, attracting interest in understanding their biosynthetic pathways, physiological roles, and involvement in disease processes. However, the mechanisms underlying LD biogenesis and function, as well as their associations with human diseases, remain poorly understood [28]. Although various reagents exist for labeling LDs, LD formation requires extended time, highlighting the need for label‐free observation methods to circumvent phototoxicity and photobleaching limitations.

**Fig. 7:**
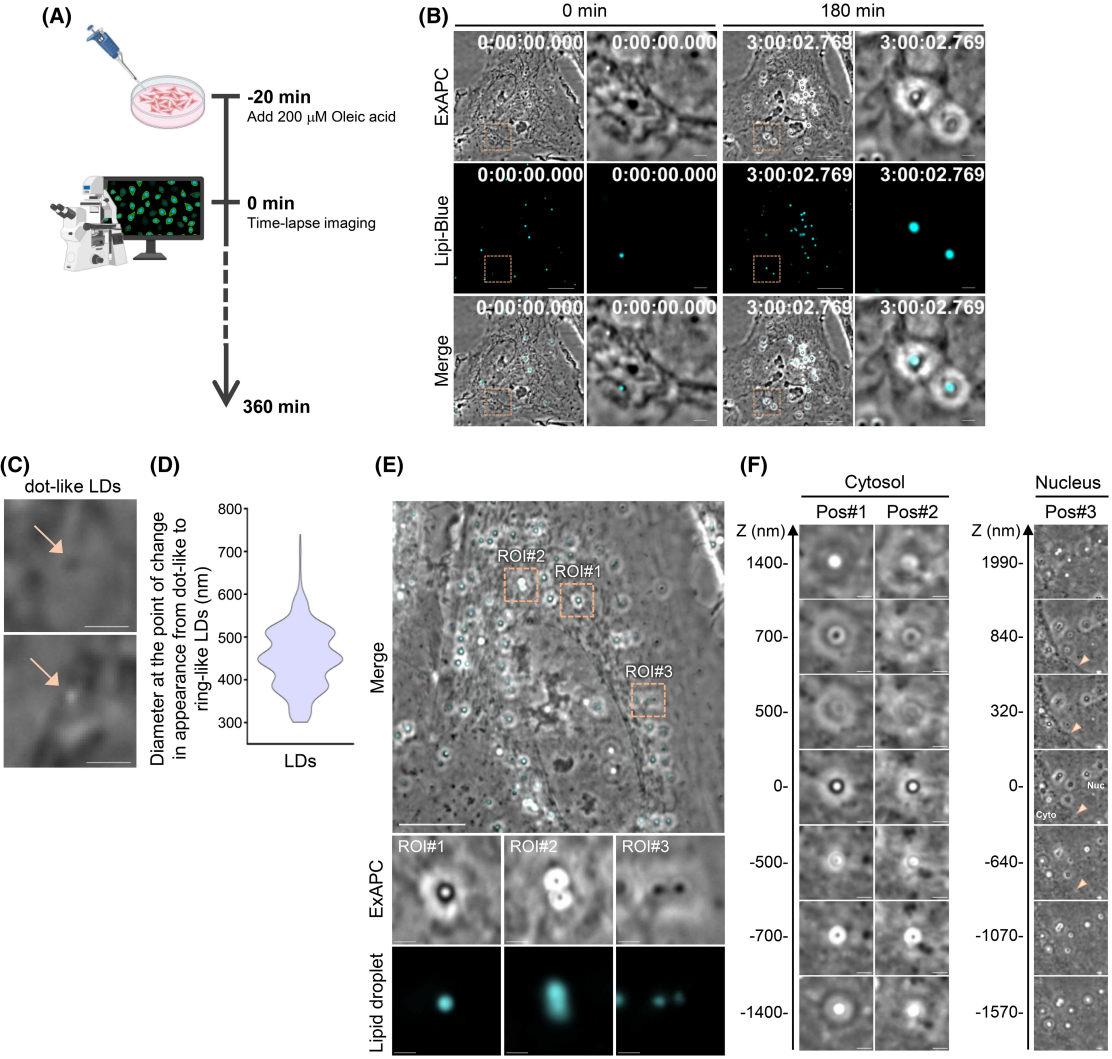
Visibility of LDs using ExAPC microscopy. (A) Schematic diagram of this experiment. Illustration created with BioRender.com. (B) Oleic acid treatment‐induced lipid droplet (LD) formation. HeLa cells were treated with oleic acid. Scale bar: 10 μm; scale bar in inset: 1 μm. (C) Representative images of LDs in Phase 1. Arrows indicate LDs. Scale bar: 1 μm. (D) Diameter at the point of change in appearance from dot‐like to ring‐like LDs (*n* = 165). (E) Representative images of LDs in Phases 2 and 3 in oleic acid‐treated HeLa cells. Scale bar: 10 μm; scale bar in inset: 1 μm. (F) The visibility of LDs according to the difference in focal planes. Scale bar: 1 μm. Time: h:mm:ss.ms. Representative images shown in (B), (C), (E), and (F) are from three independent experiments.

We acquired time‐lapse images of HeLa cells treated with 200 μm oleic acid to observe LD biosynthesis in detail using ExAPC microscopy (Fig. 7A). Among the intracellular structures observed, those stained with Lipi‐Blue, which enhances fluorescence in hydrophobic environments [29], were identified as LDs (Fig. 7B). Consequently, ExAPC microscopy enabled us to trace the full process of LD growth, including their initial appearance, which is often difficult to capture with Lipi‐Blue staining (Movie S8). When observed by ExAPC microscopy, LDs with a diameter less than 450.4 ± 70.9 nm appeared as white or black dot‐like structures (Fig. 7C,D). In contrast, once the LDs exceeded this diameter, they transitioned from dot‐like to ring‐like structures (Fig. 7E). Notably, the appearance of ring‐like LDs varied depending on focal position (Fig. 7F).

**Fig. 8:**
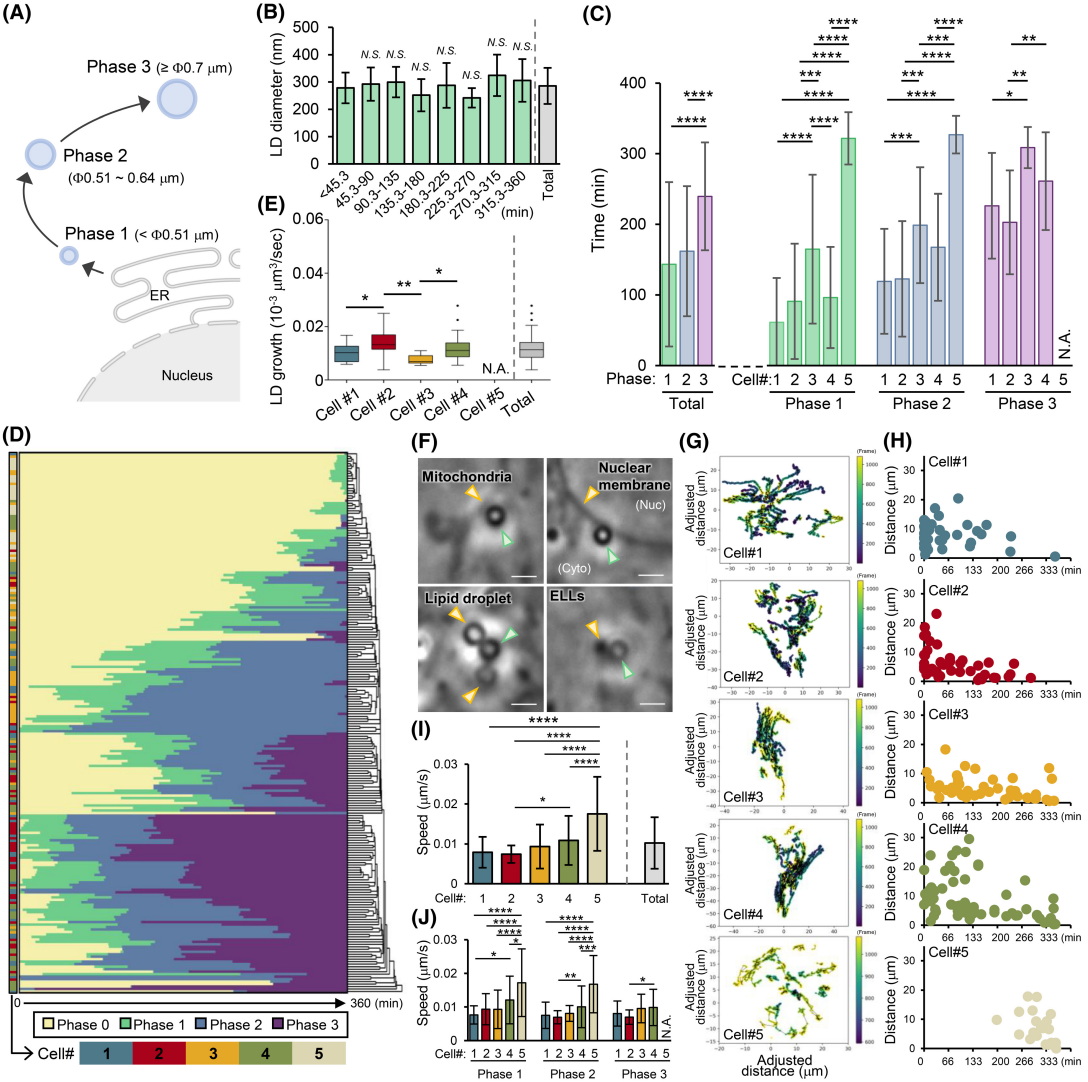
Visualization of LD dynamics using ExAPC microscopy. (A) Growth process of LDs. Illustration created with BioRender.com. (B) Diameters of LDs recognized as Phase 1 at each time point after image acquisition began (frame 1: 0 min). Acquisition interval: 20 s·frame^−1^. Means ± standard deviation. Number of LDs at each period from left to right: 51, 21, 29, 15, 10, 14, 12, 24. Statistical significance was determined by one‐way ANOVA followed by Dunnett's multiple comparisons test against the <45.3 group. NS, no significant difference. Total: all analyzed LDs (*n* = 176). (C) Time for each LD to reach each phase defined by diameter (see A). Mean ± standard deviation. [Total] Phase 1: *n* = 176, Phase 2: *n* = 162, Phase 3: 123, [Phase 1] Cell#1: *n* = 27, Cell#2: *n* = 36, Cell#3: *n* = 42, Cell#4: *n* = 42, Cell#5: *n* = 29, [Phase 2] Cell#1: *n* = 33, Cell#2: *n* = 42, Cell#3: *n* = 36, Cell#4: *n* = 42, Cell#5: *n* = 9, [Phase 3] Cell#1: *n* = 25, Cell#2: *n* = 37, Cell#3: *n* = 8, Cell#4: *n* = 53, Cell#5: *n* = 0. Statistical significance was determined by one‐way ANOVA followed by Tukey's multiple comparisons: between phases within the ‘Total’ group, and between cells within each of the Phase 1–3 groups. (D) Heatmap of phase transitions for each LD (*n* = 218). The cells analyzed (Cell#1–5) and in each phase (Phase 0–3) are color coded as indicated. The dendrogram shows hierarchical clustering based on the time at which the phase transition occurred for each LD. (E) LD growth rate based on the Phase 2–3 transition time. The numbers of LDs analyzed were as follows: Cell#1: *n* = 23; Cell#2: *n* = 35; Cell#3: *n* = 8; Cell#4: *n* = 35; Cell#5: *n* = 0 (*N*.A.); and Total: *n* = 101. One‐way ANOVA for multiple comparisons was performed between each cell. Statistical significance was determined by one‐way ANOVA followed by Tukey's multiple comparisons between cells. *P < 0.05, **P < 0.01. (F) Interaction of LDs with various organelles in HeLa cells. Green arrowhead: LD. Yellow arrowhead: organelle interacting with the LD. Scale bar: 1 μm. ELL, endosome‐ and lysosome‐like structure. (G) LD dynamics. The coordinates of LDs are corrected for the coordinates of nucleoli in the same cell. The time at which the coordinates of each LD were obtained after the addition of oleic acid is color coded as indicated. The number of LDs analyzed in each cell is as follows: Cell#1: *n* = 36; Cell#2: *n* = 44; Cell#3: *n* = 44; Cell#4: *n* = 65; and Cell#5: *n* = 28. (H) Euclidean distance between the coordinates at which each LD was first identified and the coordinates at the end of time‐lapse imaging. The number of plotted LDs is the same as that in G. (I) LD migration velocity in each cell (Cell #1–5) and in all cells (total). Means ± standard deviation. The analyzed LDs is the same as that in G. Statistical significance was determined by one‐way ANOVA followed by Tukey's multiple comparisons between cells. *P < 0.05, ****P < 0.0001. (J) LD migration velocity in each cell (Cell#1–5) in each phase. Means ± standard deviation. The analyzed LDs is the same as that in G. The numbers of LDs analyzed were as follows: [Phase 1] Cell#1: *n* = 33, Cell#2: *n* = 42, Cell#3: *n* = 42, Cell#4: *n* = 43, Cell#5: *n* = 28, [Phase 2] Cell#1: *n* = 36, Cell#2: *n* = 44, Cell#3: *n* = 38, Cell#4: *n* = 58, Cell#5: *n* = 10, [Phase 3] Cell#1: *n* = 25, Cell#2: *n* = 37, Cell#3: *n* = 8, Cell#4: *n* = 53, Cell#5: *n* = 0. Statistical significance was determined by one‐way ANOVA followed by Tukey's multiple comparisons between cells within each of the Phase 1–3 groups. *P < 0.05, **P < 0.01, ***P < 0.001, ****P < 0.0001.

To analyze the formation and growth process of LDs, we defined the period during which no LDs were observed as phase 0 and subsequently categorized phases 1 to 3 based on LD diameter (Fig. 8A). When the 218 LDs that were positive for Lipi‐Blue in the final frame were analyzed, 176 LDs appeared as phase 1 LDs during time‐lapse imaging, with a mean diameter of 285.6 ± 66.2 nm (Fig. 8B). This diameter is similar to that of pre‐LDs (257.6 ± 31 nm), an ER microdomain containing a stable core of neutral lipids in early LD biogenesis [30]. The time required for LDs to transition to phases 1, 2, and 3 was 143.3 ± 116.4 min (*n* = 176), 161.9 ± 92.1 min (*n* = 162), and 239.6 ± 76.4 min (*n* = 123), respectively, after imaging began (Fig. 8C). The mean transition times varied significantly among cells (Fig. 8C). The growth behavior of LDs after oleic acid addition also varied among cells (Fig. 8D). The time for LDs to grow from phase 2 to phase 3 was 110.3 ± 44.0 min (*n* = 101), yielding an average growth rate of 0.012 ± 0.004 × 10^−3^ μm^3^·s^−1^ (Fig. 8E). This growth rate also varied significantly among cells (Fig. 8E). Notably, no LD size changes due to fusion or division were observed during the imaging period.

LDs frequently interact with organelles, serving as lipid sources for energy production and signal transduction [31]. Using ExAPC microscopy, we observed interactions between LDs and various organelles, including mitochondria and the nucleus, without staining the target organelles (Fig. 8F). Additionally, by tracking LD dynamics, we found that LDs moved extensively throughout the cell as they grew (Fig. 8G,H). The migration velocity of LDs was 0.01 ± 0.006 μm·s^−1^, with statistically significant variation between cells (Fig. 8I,J).

Taken together, these observations highlight the complex behavior and dynamic nature of LDs within the cellular environment, including their interactions with other organelles and spatial redistribution across different cellular compartments.

### Mitochondrial dynamics analyzed using ExAPC microscopy

**Fig. 9:**
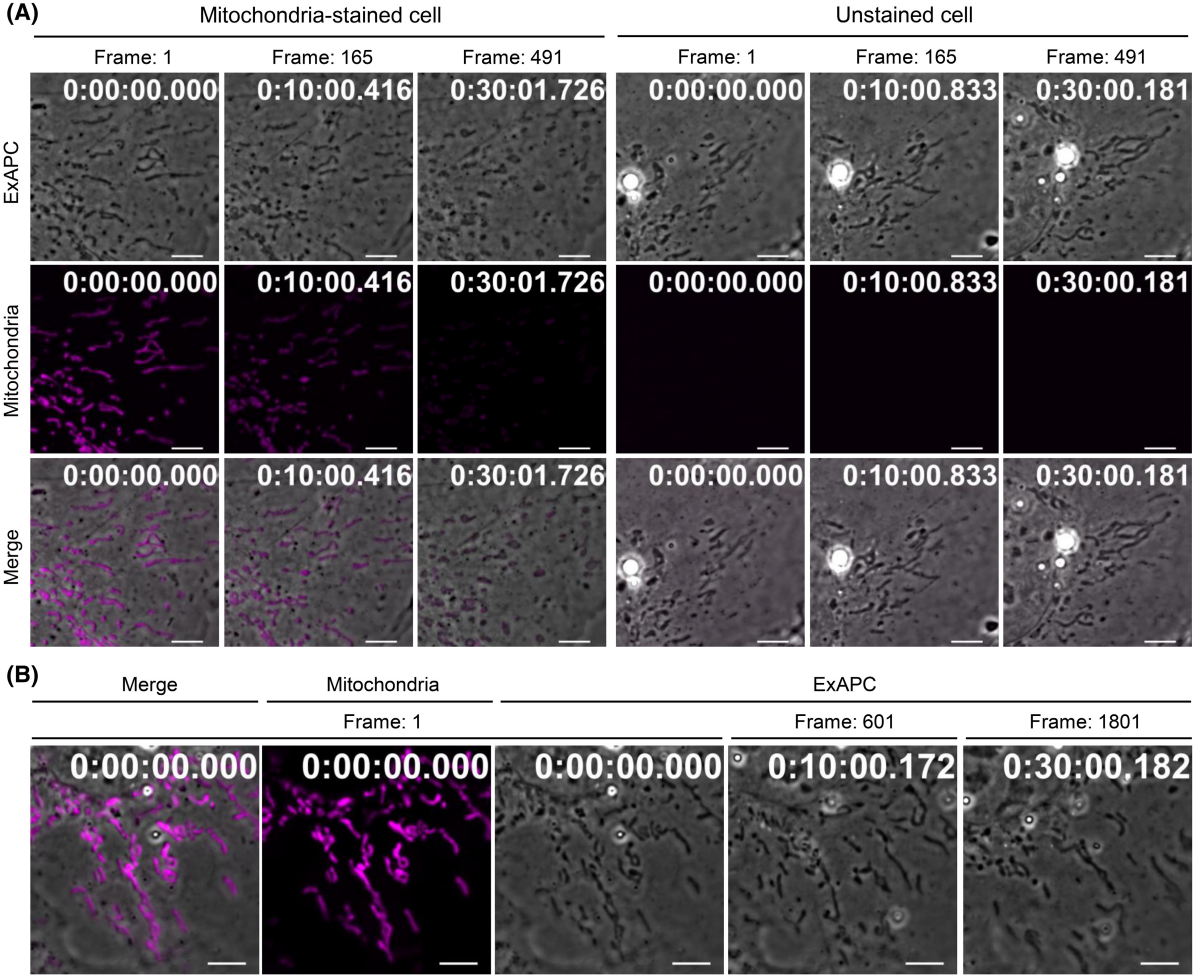
Phototoxicity and photobleaching in fluorescence imaging. (A) Hep3B cells with or without MitoTracker Red CMXRos staining were subjected to alternating and continuous acquisition (491 frames/30 min) using ExAPC and fluorescence microscopy. (B) Fluoro‐Guided ExAPC Imaging. 1801 frames/30 min. Scale bar: 5 μm. Time: h:mm:ss.ms. Representative images shown in (A) and (B) are from three independent experiments.

Mitochondria are dynamic organelles that undergo tightly regulated fission and fusion events [32]. Disruption of these regulatory mechanisms has been linked to various diseases, including cancer, cardiovascular diseases, and neurodegenerative disorders [33]. Therefore, observing mitochondrial morphology is crucial for clarifying pathogenetic mechanisms. However, assessing mitochondrial morphology through fluorescence imaging requires mitochondria‐specific probes and precise imaging parameters, such as light intensity and exposure time [34]. Indeed, when mitochondria are stained with the commonly used probe and subjected to continuous fluorescence imaging, pronounced photobleaching is frequently observed (Fig. 9A; Movie S9). In addition, phototoxic effects induced by repeated fluorescence excitation lead to progressive fragmentation of the mitochondrial network over time (Fig. 9A; Movie S9). Therefore, for long‐term or high‐temporal‐resolution observation of mitochondrial dynamics, we employed a hybrid imaging strategy, referred to here as Fluoro‐Guided ExAPC Imaging. In this approach, both ExAPC and fluorescence images are acquired at the initial point to identify mitochondria in the ExAPC image based on the fluorescence signal. Subsequent imaging is then performed exclusively using ExAPC microscopy. This strategy minimizes photobleaching and phototoxicity, enabling long‐term imaging with high‐temporal resolution (Fig. 9B).

**Fig. 10:**
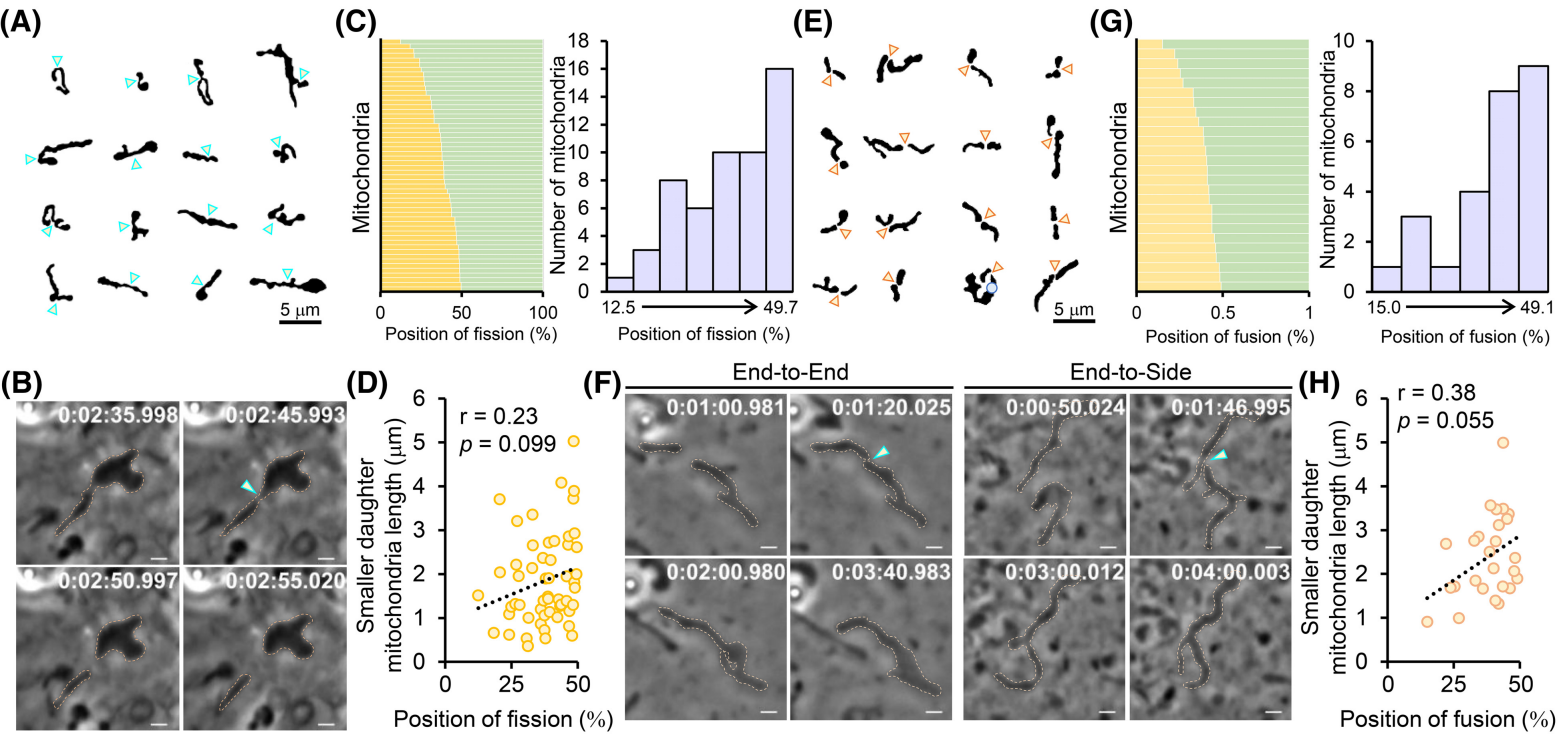
Visualization of mitochondrial dynamics using ExAPC microscopy. (A) Gallery image of mitochondria one frame before division, derived from a binarized image obtained by ExAPC microscopy. Scale bar: 5 μm. (B) Time‐lapse imaging of mitochondrial fission in Hep3B cells. Arrowheads indicate the sites where mitochondria undergo fission. Acquisition interval: 1 s·frame^−1^. Scale bar: 1 μm. (C) [Left] Position of mitochondrial fission relative to total mitochondrial length (*n* = 54 fissions from 16 cells in two independent experiments). [Right] Histogram of fission positions relative to the total mitochondrial length in the left figure. Width: 5.32. (D) Correlation between shorter daughter mitochondria length and the position of mitochondrial fission relative to total mitochondrial length. The analysis of mitochondria was identical to that in C. (E) Gallery image of mitochondria one frame before fusion, derived from a binarized image obtained by ExAPC microscopy. Scale bar: 5 μm. (F) Time‐lapse imaging of mitochondrial fusion in Hep3B cells. Arrowheads indicate the sites where mitochondria undergo fusion. Acquisition interval: 1 s·frame^−1^. Scale bar: 1 μm. (G) [Left] Position of mitochondrial fusion relative to total mitochondrial length (*n* = 26 fusions from 11 cells in two independent experiments). [Right] Histogram of fusion positions relative to the total mitochondrial length in the left figure. Width: 5.69. (H) Correlation between shorter daughter mitochondria length and the position of mitochondrial fusion relative to total mitochondrial length. The analysis of mitochondria was identical to that in G. Time: h:mm:ss.ms.

Leveraging the Fluoro‐Guided ExAPC Imaging, we examined spontaneous mitochondrial fission and fusion processes without pharmacological or genetic induction. This approach allowed us to precisely delineate fission sites in mitochondria of various shapes and lengths (Fig. 10A). Mitochondrial fission is driven by the cooperative action of the ER and dynamin‐related protein 1 (DRP1) in constricting mitochondrial cleavage sites [35, 36]. This constriction was detectable via ExAPC microscopy (Fig. 10B; Movie S10). The fission sites were observed at various locations along the mitochondria, with the distribution displaying a bimodal pattern (Fig. 10C). This observation supports the existence of two distinct mitochondrial fission mechanisms, as previously reported [37]. We further examined whether mitochondrial length influenced fission site location but found no significant correlation between mitochondrial length and the fission site (Fig. 10D).

The mitochondrial fusion process was similarly observed. Mitochondrial fusion consists of a two‐step process in which mitofusins (MFNs) mediate outer mitochondrial membrane fusion, and optic atrophy‐1 (OPA1) regulates inner mitochondrial membrane fusion in mammalian cells [38]. Typically, mitochondrial fusion occurs at the ends of both mitochondria, although end‐to‐side fusion can occur in rare instances [39] (Fig. 10E,F; Movies S11, S12). Similar to fission, a gradual bimodal distribution was observed for the relative lengths of the two fused mitochondria (Fig. 10G). However, the relative length of the smaller mitochondria was greater than 30% in 80.7% of fusion events, indicating that mitochondria of similar length tended to fuse (Fig. 10G). As with fission, we found no substantial correlation between mitochondrial length and fusion site (Fig. 10H). These findings suggest that the ratio of mitochondrial lengths should be considered when examining the mechanisms governing mitochondrial fission and fusion.

**Fig. 11:**
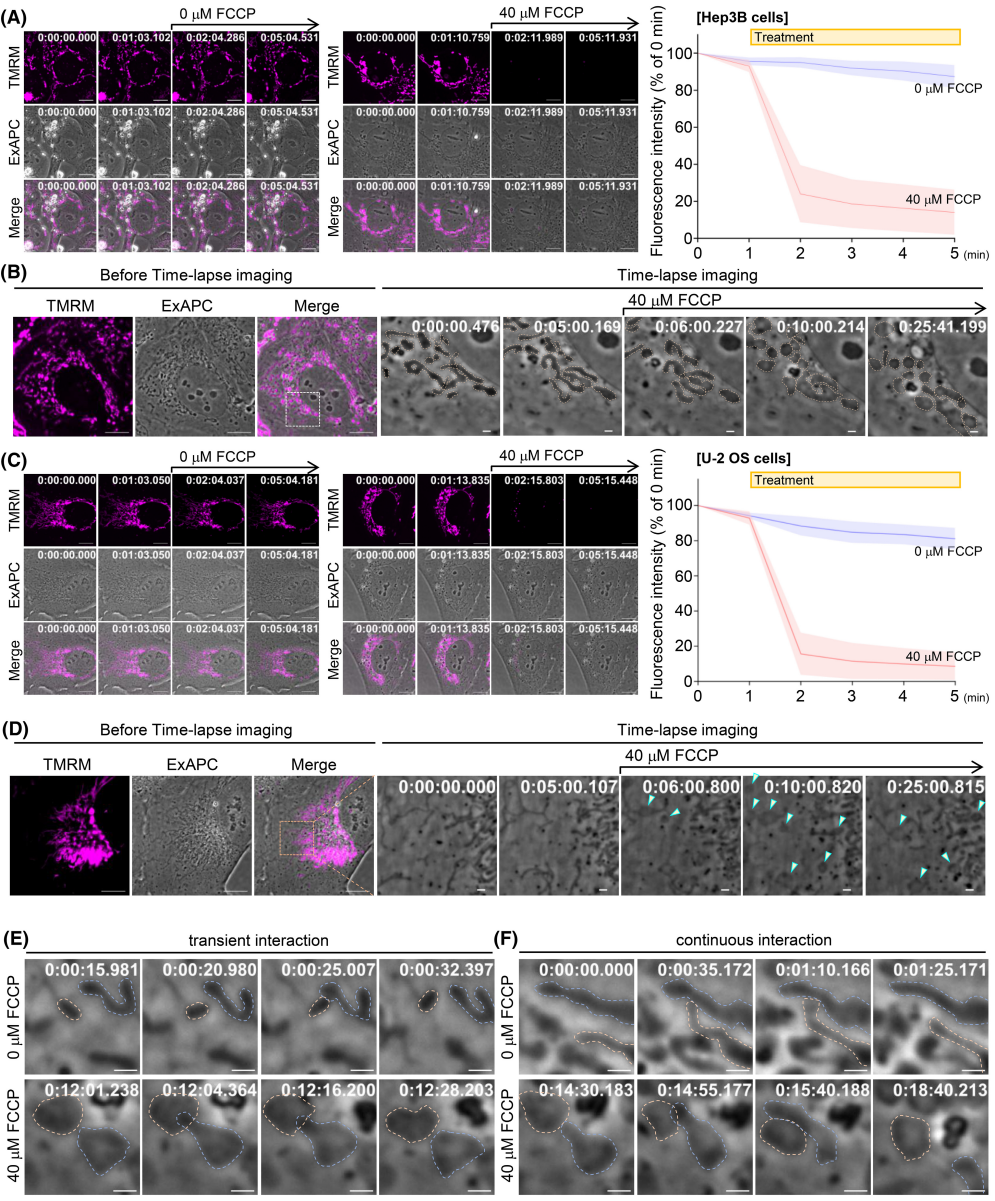
FCCP‐Induced mitochondrial morphological changes visualized by ExAPC microscopy. (A) Mitochondrial membrane potential in Hep3B cells. Representative images at each time point are shown. ExAPC and tetramethylrhodamine methyl ester (TMRM) fluorescence images were sequentially acquired at each frame. All the data are presented as the mean ± standard deviation. 0 μm carbonylcyanide‐p‐trifluoromethoxyphenylhydrazone (FCCP): *n* = 14 cells. 40 μm FCCP: *n* = 49 cells. Acquisition interval: 1 min·frame^−1^. Scale bar: 10 μm. (B) FCCP treatment‐induced changes in mitochondrial morphology in Hep3B cells. Images were acquired using the Fluoro‐Guided ExAPC Imaging method. FCCP was added after image acquisition at frame 301. Acquisition interval: 1 s·frame^−1^. Scale bar: (Before Time‐lapse imaging): 10 μm, (Time‐lapse imaging): 1 μm. (C) Mitochondrial membrane potential in U‐2 OS cells. Representative images at each time point are shown. ExAPC and TMRM fluorescence images were sequentially acquired at each frame. All the data are presented as the mean ± standard deviation. 0 μm FCCP: *n* = 9 cells. 40 μm FCCP: *n* = 30 cells. Acquisition interval: 1 min·frame^−1^. Scale bar: 10 μm. (D) FCCP treatment‐induced changes in mitochondrial morphology in U‐2 OS cells. Images were acquired using the Fluoro‐Guided ExAPC Imaging method. FCCP was added after image acquisition at frame 301. Arrowheads indicate portions of mitochondria that have become swollen and adopted a globular morphology following FCCP treatment. Acquisition interval: 1 s·frame^−1^. Scale bar: (Before Time‐lapse imaging): 10 μm, (Time‐lapse imaging): 1 μm. (E, F) Intermitochondrial communication in the presence or absence of FCCP in Hep3B cells. Orange dashed outlines indicate one mitochondrion, and blue dashed outlines indicate another mitochondrion involved in the communication event. Scale bar: 1 μm. Acquisition interval: 1 s·frame^−1^. Time: h:mm:ss.ms.

Exposure to an oxidative phosphorylation uncoupler, such as carbonylcyanide‐p‐trifluoromethoxyphenylhydrazone (FCCP), leads to a reduction in mitochondrial membrane potential (ΔΨm) and induces reactive oxygen species (ROS) stress within the cell, causing mitochondria to transform from elongated string‐like structures into small globular spaces [40]. However, the characteristics of mitochondrial morphology following ΔΨm loss remain debated. To investigate these features, we treated Hep3B and U‐2 OS cells with 40 μm FCCP to induce a substantial decrease in ΔΨm and monitored the resulting changes in mitochondrial morphology. In Hep3B cells, individual mitochondria rapidly transitioned from filamentous to globular forms within a few minutes (Fig. 11A,B; Movie S13). In contrast, in U‐2 OS cells, rather than the entire mitochondrion becoming spherical, only portions of the mitochondria adopted a globular morphology (Fig. 11C,D; Movie S14). Furthermore, these morphological changes varied between individual mitochondria in both Hep3B and U‐2 OS cells (Fig. 11B,D). These results suggest heterogeneity in mitochondrial responses to FCCP.

Mitochondria are known for their ability to communicate through physical contact [41]. We observed this intermitochondrial communication using ExAPC microscopy, both under normal culture conditions and in conditions where ΔΨm was disrupted by FCCP treatment (Fig. 11E,F). These findings suggest that intermitochondrial communication can occur independently of ΔΨm.

## Discussion

In this study, we demonstrate that ExAPC microscopy is a valuable tool for examining cellular organization in diverse cell types, such as human cancer cell lines, primary cultured cells, and iPS cells. With its high spatiotemporal resolution and multiplexing capabilities, this label‐free imaging modality facilitates simultaneous visualization of various intracellular structures and allows detailed analysis of morphological changes in cellular organization across a range of physiological and pathological contexts. Despite recent advances in photostable fluorescent proteins [42, 43], label‐free imaging provides distinct advantages, particularly enabling prolonged observations of living cells with minimal concerns about phototoxicity and photobleaching. In addition, it helps to avoid undesirable effects caused by staining reagents and transfection agents, as well as morphological abnormalities that may arise from the overexpression of organelle‐targeted fluorescent proteins [44].

ExAPC microscopy still has several limitations. In particular, halo artifacts are frequently observed around thick cells and specific intracellular structures (e.g., mature lipid droplets), which can hinder accurate segmentation and quantitative analysis. In this study, we did not apply any postprocessing for halo removal; however, the integration of computational techniques—such as spatial filtering or deep learning‐based correction—may further improve segmentation accuracy and analytical robustness in future applications.

The effective imaging depth of ExAPC microscopy is influenced not only by the physical thickness of the specimen but also by internal refractive index heterogeneity. For instance, visualizing intracellular structures within organoids or *ex vivo* tissue slices remains technically challenging with the current system, indicating that ExAPC is presently best suited for single‐layer or moderately thick samples. Advances in phase reconstruction algorithms and axial optical sectioning techniques hold the potential to overcome these depth‐related limitations, thereby enabling the application of ExAPC to thicker and more structurally complex specimens—ultimately paving the way for its future use in *in vivo* imaging.

Furthermore, many intracellular components cannot be reliably identified by ExAPC alone, highlighting the need for integration with complementary imaging modalities such as fluorescence microscopy. Recent advances in computational imaging, including digital staining and deep learning‐based approaches, offer promising solutions to these limitations. By combining ExAPC with such computational methods, molecular specificity and structural recognition can be significantly enhanced, potentially enabling comprehensive, label‐free analysis of diverse cellular features.

We obtained quantitative data on biomolecular condensates, lipid droplets, and mitochondria using ExAPC microscopy, and comparable results can be achieved using conventional fluorescence imaging, except for BCLs. Therefore, ExAPC microscopy offers a significant advantage in its label‐free imaging capability, enhancing experimental flexibility. For instance, in label‐free imaging, there is no need to account for the potential effects of staining on the cells, allowing for subsequent quantification of gene or protein expression levels after observation using ExAPC microscopy. Moreover, the reduced risk of phototoxicity and photobleaching in ExAPC microscopy enables extended real‐time observation of cellular dynamics, which is a major benefit. Selecting the optimal microscopic technique for specific experimental goals is critical for advancing science research. ExAPC microscopy is well‐suited for experiments requiring high‐temporal resolution or the observation of thin‐cell specimens, while ODT is more effective for thicker samples or when temporal resolution is less critical. Fluorescence imaging remains indispensable for studying organelles, such as the Golgi apparatus, or specific protein activities that cannot be observed using ExAPC microscopy or ODT. Combining these techniques offers new insights into cellular structure and function, potentially driving significant advances in life science research.

Biomolecular condensates are membrane‐free intracellular compartments that orchestrate diverse biochemical reactions with spatial and temporal precision [45, 46]. However, critical uncertainties remain regarding the processes that govern their formation and the resulting modifications to biomolecule functionality. Using ExAPC microscopy, we observed BCLs for the first time. Further research is needed to elucidate the composition and function of BCLs, but their observation using ExAPC microscopy is expected to enhance our understanding of biomolecular condensates. Additionally, we discovered that the refractive index of IRS‐1 droplets varies, suggesting that these droplets consist of distinct molecular components. These findings, which are challenging to obtain using fluorescence imaging alone, highlight the importance of combining ExAPC microscopy with fluorescence‐based techniques. Over the past decade, researchers have sought to elucidate the role of biomolecular condensates in diseases such as neurodegeneration and cancer [47, 48, 49]. It may be crucial to consider the heterogeneity of biomolecular condensates in future research.

Intracellular LDs are conserved organelles present in all eukaryotic cells, storing lipids such as triacylglycerols (TAGs) and cholesterol esters while protecting cells from lipotoxicity by sequestering fatty acids [50, 51]. LDs form when TAGs accumulate in the ER membrane and bud into the cytosol, where they grow through fusion or induce *de novo* TAG synthesis. Fusion involves transferring neutral lipids from smaller to larger LDs via proteins, such as FSP27 and perilipin1, though perilipin1 [52] is mainly found in specific tissues and not in HeLa cells [53, 54]. Therefore, in our study, although we observed adjacent LDs over a long period of time, we did not detect any fusion events between them.

Early LDs are known for their short lifespan and small size, which often pose challenges to their visualization [51]. Previous studies using electron microscopy have shown that the diameter of nascent LDs varies, with studies on yeast indicating a diameter of 30–60 nm [55], and studies on mammalian cell lines a diameter of approximately 170 nm [56]. Notably, when ExAPC microscopy was employed to investigate the nascent stages of LD biogenesis, the diameter of newly formed LDs was measured at 285.6 ± 66.2 nm. Observing these early LDs requires high‐magnification objectives, and the ExAPC method is well‐suited for this purpose owing to the capacity to select appropriate lenses. Furthermore, in our study, the growth rate of LDs was calculated to be 0.012 ± 0.004 × 10^−3^ μm^3^·s^−1^, a growth rate closer to that observed in COS‐1 cells (0.043 × 10^−3^ μm^3^·s^−1^) [30] than to the rate observed in Drosophila (0.159 × 10^−3^ μm^3^·s^−1^) [57]. Thus, label‐free imaging using the ExAPC microscope is a powerful tool for quantitatively analyzing the biogenesis of LDs and their spatial relationships with other intracellular structures.

Mitochondria are multifunctional organelles that undergo dynamic morphological changes through repeated fission and fusion processes [58]. The mitochondria exhibit a plethora of distinct morphologies within different cells, ranging from reticulation throughout the cell to fragmentation into discrete pieces [59]. A series of distinctive morphological alterations in mitochondria have been described during processes such as stem cell differentiation and multifactorial diseases, such as cancer, cardiovascular diseases, and neurodegenerative diseases [5, 60, 61]. These observations highlight the significance of mitochondrial morphology in various physiological and pathological processes. Understanding the regulatory mechanisms governing mitochondrial fission and fusion is crucial, as these processes determine mitochondrial morphology. In this study, specific patterns were observed regarding the sites of mitochondrial fission and the lengths of two mitochondria undergoing fusion. While the underlying mechanisms governing these regularities remain elusive, mitochondria may possess an overarching ability to perceive their own length and that of neighboring mitochondria, potentially guiding these processes. We also found that ΔΨm loss leads to distinct morphological changes, with Hep3B mitochondria becoming spherical, while U‐2 OS mitochondria displayed partial spherical morphology. This discrepancy may stem from differences in mitochondrial lipid composition. In addition, we found that individual mitochondria responded differently to FCCP treatment, suggesting that heterogeneity in mitochondrial responsiveness may play a role in regulating overall mitochondrial function. This variability in cellular responses has been reported to enable more precise control of tissue responses [62], and similar regulatory systems may exist within mitochondria. Further investigations are required to address these questions. We believe that ExAPC microscopy data will stimulate further exploration of these biological questions and contribute to a deeper understanding of mitochondrial biology.

## Materials and methods

### Plasmid construction

EYFP‐Cb5 was produced by subcloning the sequence coding aa 100–134 of rat Cytochrome b5 (UniProtKB‐P00173) into an EYFP vector at the C terminus between the EcoR I and Sal I sites. EYFP‐sGOLGB1 was produced by subcloning the sequence encoding aa 3131–3259 of human GOLGB1 (UniProtKB‐Q14789) into an EYFP vector at the C terminus between the EcoR I and Sal I sites. Lifeact‐EYFP was kindly provided by Dr. Hideki Nakamura (Kyoto University). GFP‐rIRS‐1 was produced by subcloning the sequence coding aa 4–1235 of rat IRS‐1 (UniProtKB‐P35570) into an EGFP vector at the C terminus between Hind III and the BamH I site.

### Cell culture and transfection

Human lung carcinoma A549 cells (RRID:CVCL_0023, ATCC CCL‐185), human cervical adenocarcinoma HeLa cells (RRID:CVCL_0030, ATCC CCL‐2), human colon carcinoma HCT116 cells (RRID:CVCL_0291, ATCC CCL‐247), human hepatocellular carcinoma Hep 3B cells (RRID:CVCL_0326, ATCC HB‐8064), mouse fibroblast NIH/3T3 cells (RRID:CVCL_0594, ATCC CRL‐1658), human breast adenocarcinoma MDA‐MB‐231 cells (RRID:CVCL_0062, ATCC CRM‐HTB‐26), human osteosarcoma U2‐OS cells (RRID:CVCL_0042, ATCC HTB‐96), and human breast carcinoma ZR75‐1‐1 (RRID:CVCL_0588, ATCC CRL‐1500) cells were purchased from the American Type Culture Collection (ATCC, Manassas, VA, USA). All cell lines were originally authenticated by ATCC. Mycoplasma contamination was routinely tested using the e‐Myco™ Mycoplasma PCR Detection Kit (#25235, iNtRON Biotechnology, Seongnam, Korea), and all experiments were performed with mycoplasma‐free cells. These cells, except ZR75‐1‐1, were cultured in Dulbecco's modified Eagle's medium (DMEM; Thermo Fisher Scientific, Waltham, MA, USA, 11965118) supplemented with 10% fetal bovine serum (FBS; Thermo Fisher Scientific, 10 270‐106) and 1% Zell Shield (Minerva Biolabs GmbH, Berlin, Germany, 13–0050) at 37 °C in 5% CO_2_. ZR75‐1‐1 cells were cultured in RPMI 1640 medium (Thermo Fisher Scientific, 11 875 093) supplemented with 10% FBS and 1% Zell Shield at 37 °C in 5% CO_2_. For transient transfection, 2 × 10^5^ cells were plated on a 35 mm imaging dish with a polymer coverslip bottom (ibidi GmbH, Gräfelfing, Germany, 80 136) and incubated for 6 h at 37 °C in 5% CO_2_. Following incubation, the cells were transfected with the plasmid using FuGENE HD (Promega Corporation, Madison, WI, USA, E2311). The indicated experiments were carried out 12–18 h after transfection. The TKT3V1–7 iPSC clone was generated from peripheral blood T cells by Sendai virus reprogramming vectors as previously described [63]. The iPSC line was provided by CiRA with the approval of the Ethical Review Board of CiRA (no. G590). iPSCs were maintained on an iMatrix‐511 (Matrixome Inc., Osaka, Japan) as previously described [64], but StemFit AK02N (Ajinomoto Healthy Supply Co., Inc., Tokyo, Japan) was used instead of AK03. The experimental protocol for TKT3V1–7 was approved by the institutional regulation board for human ethics at the Institute of Medical Science, University of Tokyo (approval number: 20‐6‐0826). All experiments were conducted in accordance with the standards set by the Declaration of Helsinki. The study was performed with the understanding and written informed consent from whom the sample was collected. The sample was collected at the Institute of Medical Science, University of Tokyo.

### Animals

The research protocol was approved by the Animal Care Committee, University of Tsukuba (Approval number: 24‐097), and all experimental procedures involving animals were conducted according to the guidelines. C57BL/6 mice were purchased from CLEA Japan, Inc and maintained on a 14‐h light and 10‐h dark period cycle with free access to water and a standard chow diet. To obtain primary cultures, mating was performed using 8‐week‐old male and female mice.

### Primary astrocyte and neuron culture

Primary culture was performed as previously described [65, 66], with slight modifications. Astrocytes were prepared from the hippocampus on postnatal day 2 and neurons on embryonic day 17.5. Hippocampal extraction was performed as previously described [67]. Cells were isolated using Neural Tissue Dissociation Kits (Miltenyi Biotec, Bergisch Gladbach, Germany, 130‐093‐231). Astrocytes were then seeded at 5 × 10^5^ cells in 25 mm^2^ flasks (VIOLAMO, Osaka, Japan, 2‐8589‐01) coated with poly‐L‐lysine (Merck KGaA, Darmstadt, Germany, P4707), and after 7 days, cells were detached by trypsin treatment and seeded at 3 × 10^5^ cells in 35 mm imaging dishes with polymer coverslip bottoms coated with poly‐l‐lysine and cultured for 7 days. Neurons were seeded at 5 × 10^5^ cells in 35 mm imaging dishes with a polymer coverslip bottom coated with poly‐L‐lysine and cultured for 10 days. Astrocytes were cultured in Dulbecco's modified Eagle's medium (DMEM) supplemented with 10% fetal bovine serum and 1% penicillin–streptomycin (Nacalai Tesque, Inc., Kyoto, Japan, 09367‐34) at 37 °C in 5% CO_2_. Neurons were cultured in a Neurobasal medium (Thermo Fisher Scientific, 21 103 049) supplemented with 1% B‐27 (Thermo Fisher Scientific, 17 504 044), 1% GlutaMAX (Thermo Fisher Scientific, 35 050 061), and 1% penicillin–streptomycin at 37 °C in 5% CO_2_.

### Construction of an ExAPC microscopy system

To construct an ExAPC microscopy system, the apodized phase plate—normally integrated at the pupil plane within conventional phase‐contrast objectives—was relocated to an optically conjugate plane. In the Ti2‐E inverted microscope (Nikon, Tokyo, Japan), this conjugate plane lies within the base of the optical tube. Accordingly, a motorized external phase‐contrast tube base (TI2‐T‐BP‐E, Nikon) was implemented, and the apodized phase plate (TI2‐T‐XPH‐PH4 100×, Nikon) was mounted at this location. The imaging setup employed an Apo TIRF 100× objective lens (CFI Apochromat TIRF 100XC Oil, NA = 1.49, Nikon), in conjunction with a CLWD condenser lens (TI‐C, Nikon) and the corresponding phase annulus Ph4 (TI2‐C‐MC‐PH4, Nikon) to ensure compatibility with the optical path. For optical alignment, the eyepiece was removed and replaced with a centering telescope, allowing direct observation of the conjugate pupil plane. The apodized phase plate includes mechanisms for fine adjustment of both centering and focal position. These were tuned so that the condenser‐side ring slit image coincided optically with the apodized phase plate. Upon completion of the alignment, the telescope was replaced with the eyepiece, allowing for image acquisition via the externally mounted apodized phase plate. The tube base also includes a camera port and a switchable mirror. By toggling the mirror toward the camera path, external phase‐contrast images could be captured.

### Live‐cell imaging

For ExAPC microscopy, ExAPC images were acquired using an Eclipse Ti2‐E (Nikon) microscope and a Zyla‐4.2P monochrome CMOS camera (Andor Technology Ltd., Belfast, Northern Ireland, UK), and were obtained using an oil immersion lens (CFI Apochromat TIRF 100XC Oil, NA = 1.49, Nikon). Based on this optical configuration and assuming a center illumination wavelength of 550 nm and using immersion oil with a refractive index of 1.518, the theoretical lateral and axial resolutions of the ExAPC system are approximately 250 and 450 nm, respectively. The average illumination power density (irradiance) at the specimen plane was estimated to be approximately 73 μW·mm^−2^, based on illuminance measurements obtained using a calibrated lux meter placed at the sample position.

For ODT, an inverted Eclipse Ti2‐E microscope (Nikon) was used, and bright‐field images were acquired using a Digital Sight 50 M monochrome CMOS camera (Nikon). Bright‐field images were obtained using an oil immersion lens (CFI Plan Apochromat Lambda 100XH, NA = 1.45, Nikon). Based on this optical configuration and assuming a center illumination wavelength of 550 nm and using immersion oil with a refractive index of 1.518, the theoretical lateral and axial resolutions of the ExAPC system are approximately 280 and 510 nm, respectively. Imaging data were processed according to previously reported formula [68]. It should be noted that the actual resolution of both the ExAPC and ODT systems is dependent on the optical configuration, including the numerical aperture of the objective lens, illumination wavelength, and the refractive index of the immersion oil. Any change in these parameters may result in a proportional shift in the theoretical resolution. Regarding objective lens selection, the CFI Plan Apochromat Lambda 100XH objective lens (NA 1.45) was selected for use in the ODT imaging system because it offers excellent field flatness even at the periphery of the field of view, making it particularly suitable for wide‐field imaging. In contrast, the CFI Apochromat TIRF 100XC Oil objective lens (NA 1.49), which was used for ExAPC microscopy, provides a higher numerical aperture but does not ensure sufficient image flatness at the edges. While it performs adequately when used via a camera port in applications with a limited field number—such as in ExAPC microscopy—it is less suitable for wide‐field acquisition through a side port, as required in ODT. Thus, the NA 1.45 objective lens was chosen to achieve an optimal balance between spatial resolution and peripheral image quality in the ODT system.

For epifluorescence microscopy images, BFP, EYFP, and mCherry excitation was carried out using an Intensilight mercury‐fiber illuminator (Nikon). The data were processed through a BFP‐A‐Basic filter (Semrock, Inc., Rochester, NY, USA), a CFP‐A‐Basic‐NTE filter (Semrock), a YFP‐A‐Basic‐NTE filter (Semrock), and an mCherry‐B‐NTE‐ZERO filter (Semrock) for BFP, EYFP, and mCherry imaging, respectively. Imaging data were processed using NIS‐Elements AR imaging software (Nikon).

All imaging experiments were completed at 37 °C in 5% CO_2_ using an STX stage top incubator (Tokai Hit Co., Ltd., Fujinomiya, Japan). For all live‐cell imaging, unless otherwise noted, cells were cultured in phenol red‐free DMEM (Thermo Fisher Scientific, 31 053 028) supplemented with 10% FBS, 4 mm l‐glutamine (Thermo Fisher Scientific, 25 030 081), and 1% penicillin–streptomycin (Sigma‐Aldrich, St. Louis, MO, USA, P4333). The following representative images obtained by epifluorescence microscopy were processed by Clarify.ai and Denoise.ai using NIS‐Elements AR 5.30: mitochondria, ER, Golgi apparatus, endosome, and lysosome. The sizes of ELLs were measured using NIS‐Elements General Analysis software. Specifically, images were processed by applying local contrast enhancement (Degree: 25%, Radius: 0.96 μm) followed by thresholding (Intensity range: 0‐800; Smooth: 0.064 μm; Clean: 0.032 μm; Fill Holes: OFF; Separate: 0.13; Circularity: 0.8–1). The areas of the identified structures were subsequently measured.

### Organelle staining

A total of 2 × 10^5^ cells were plated on a 35 mm imaging dish with a polymer coverslip bottom and incubated for 12–18 h at 37 °C in 5% CO_2_. Following incubation, the cells were cultured with 0.1 μg·mL^−1^ Hoechst 33342 (Thermo Fisher Scientific, 62 249) for 10 min at 37 °C in 5% CO_2_ to stain the nuclei. For mitochondrial staining, the cells were cultured with 0.5 μm MitoTracker Red CM‐H2Xros (Thermo Fisher Scientific, M7513) for 30 min at 37 °C in 5% CO_2_. The endosome staining was performed using the ECGreen‐Endocytosis Detection kit (DOJINDO Laboratories, Kumamoto, Japan, E296) in accordance with the provided protocol. For lysosome staining, the cells were cultured with 100 nm LysoTracker Red DND‐99 (Thermo Fisher Scientific, L7528) for 30 min at 37 °C in 5% CO_2_. The cells were then washed with imaging medium twice and subjected to live‐cell imaging.

### Monitoring cell division

For the HeLa cells, 2 × 10^5^ cells were plated on a 35 mm imaging dish with a polymer coverslip bottom and incubated for 24 h at 37 °C in 5% CO_2_. For iPSCs, 1.3 × 10^5^ cells were plated on a 35 mm imaging dish with a polymer coverslip bottom at 37 °C in 5% CO_2_. After 72 h, live‐cell imaging was carried out with the iPSC culture medium.

### Monitoring apoptosis

HeLa cells (2 × 10^5^ cells) were plated on a 35 mm imaging dish with a polymer coverslip bottom and incubated for 24 h at 37 °C in 5% CO_2_. Subsequently, the cells were subjected to live‐cell imaging. Then, 1 μm staurosporine (Sigma‐Aldrich, S6942‐200UL) was added at the indicated times.

### Monitoring entosis

ZR75‐1‐1 cells (4 × 10^5^ cells) were seeded in three 6 cm dishes. Forty‐eight hours later, 4 μL of 1 mm CellTracker Green CMFDA Dye (Thermo Fisher Scientific, C7025) was added to one dish and incubated for 30 min at 37 °C in 5% CO_2_. After incubation, the cells were washed 3 times with 4 mL of PBS. The ratio of stained cells to unstained cells was adjusted to 1:2, and these cells were plated onto 35 mm imaging dishes with polymer coverslips (4 × 10^5^ cells·dish^−1^) and incubated for 48 h at 37 °C in 5% CO_2_. Subsequently, the cells were subjected to live‐cell imaging.

### Monitoring BCLs and IRS‐1 droplets

HeLa cells (2 × 10^5^ cells) were plated on a 35 mm imaging dish with a polymer coverslip bottom and incubated for 24 h at 37 °C in 5% CO_2_. Subsequently, the cells were subjected to live‐cell imaging. 1,6‐Hexanediol (Sigma‐Aldrich, 240 117) and sodium chloride (Nacalai Tesque, Inc., 31 320–05) were added at the indicated times. The perimeter, intensity, and motility of the BCLs and IRS‐1 droplets were analyzed by NIS software (Nikon).

### LD tracking and analysis

HeLa cells (2 × 10^5^ cells) were plated on a 35 mm imaging dish with a polymer coverslip bottom and incubated for 24 h at 37 °C in 5% CO_2_. The cells were stained with 0.5 μm Lipi‐Blue for 30 min according to the manufacturer's protocol. Oleic acid (200 μm; Sigma‐Aldrich, O1008) was then added to the cells 20 min before live‐cell imaging began. LD tracking analysis was performed using NIS software (Nikon), with three independent researchers conducting the analyses separately. To ensure reliability, all results were subsequently cross‐verified among the researchers. LD size classifications were defined based on reference ring‐like LDs (Fig. 7F, *Z* = 0 nm), with LDs having diameters of 0.51–0.64 μm classified as Phase 2. LDs smaller than this range were categorized as Phase 1, and those larger as Phase 3. For LDs imaged at different focal positions from the reference ring‐like LDs, the impact of focal shifts along the Z‐axis on measured LD sizes was predetermined, enabling accurate phase classification at each focal plane. Transitions between phases were independently evaluated by the three researchers, and final agreement was reached collectively. In calculating the growth rate of LDs from Phase 2 to Phase 3, only LDs that exhibited a transition from Phase 1 to Phase 3 during time‐lapse imaging were included in the calculation. Heatmap cluster analysis was performed by Origin Pro 2023. For plotting the behavior of LDs, the coordinates of the nucleolus in the same cell were subtracted from the coordinates of the LD.

### ΔΨm analysis

The cells were cultured for 30 min in the presence of 100 nm Image‐iT TMRM Reagent (TMRM; Thermo Fisher Scientific, I34361) at 37 °C in 5% CO_2_. The cells were then washed with imaging medium twice, and TMRE fluorescence in the mitochondria was recorded with NIS software (Nikon). Background fluorescence was measured in areas without mitochondria and subtracted from the fluorescence obtained from mitochondria.

### Statistical analysis

The results are presented as the means ± standard deviation. All the statistical tests were performed using GraphPad Prism (GraphPad Software, LLC, San Diego, CA, USA) or Excel (Microsoft Corporation, Redmond, WA, USA). The number of classes in the histogram is determined by the Sturgess formula.

## Conflict of interest

The authors declare no competing interests.

## Author contributions

TM conceived the project. TM designed the experiments. TM, HO, TN, KK, YO, NO, MK, LNS, MM, YT, TN, YH, YY, SIT, YM, YY, YT, MS, TM, and HS conducted experiments. TM, and HO wrote the manuscript.

## Supporting information


**Movie S1.** Sub‐second time‐lapse imaging.

**Movie S2.** Cell division (HeLa cells).

**Movie S3.** Cell division (iPSCs).

**Movie S4.** Apoptosis.

**Movie S5.** Entosis.

**Movie S6.** BCLs.

**Movie S7.** IRS‐1 droplet.

**Movie S8.** LD dynamics.

**Movie S9.** Photobleaching and phototoxicity.

**Movie S10.** Mitochondria fission.

**Movie S11.** Mitochondria fusion (end‐to‐end).

**Movie S12.** Mitochondria fusion (end‐to‐side).

**Movie S13.** FCCP treatment‐induced changes in mitochondrial morphology in Hep3B cells.

**Movie S14.** FCCP treatment‐induced changes in mitochondrial morphology in U‐2 OS cells.

## Data Availability

All data supporting the findings of this study are available in the Dryad Digital Repository at DOI: https://doi.org/10.5061/dryad.mgqnk99b1.
